# The Neuroprotective Effects of GPR4 Inhibition through the Attenuation of Caspase Mediated Apoptotic Cell Death in an MPTP Induced Mouse Model of Parkinson’s Disease

**DOI:** 10.3390/ijms22094674

**Published:** 2021-04-28

**Authors:** Md Ezazul Haque, Shofiul Azam, Mahbuba Akther, Duk-Yeon Cho, In-Su Kim, Dong-Kug Choi

**Affiliations:** 1 Department of Applied Life Science, Graduate School, BK21 Program, Konkuk University, Chungju 27478, Korea; mdezazulhaque@yahoo.com (M.E.H.); shofiul_azam@hotmail.com (S.A.); smritymahbuba@gmail.com (M.A.); ejrdus1026@naver.com (D.-Y.C.); 2 Department of Biotechnology, College of Biomedical and Health Science, Research Institute of Inflammatory Disease (RID), Konkuk University, Chungju 27478, Korea

**Keywords:** apoptosis, PARP, caspase 3, neurodegeneration, GPR4 receptor, MPTP, Parkinson’s disease

## Abstract

The proton-activated G protein-coupled receptor (GPCR) 4 (GPR4) is constitutively active at physiological pH, and GPR4 knockout protected dopaminergic neurons from caspase-dependent mitochondria-associated apoptosis. This study explored the role of GPR4 in a 1-methyl-4-phenyl-1,2,3,6-tetrahydropyridine (MPTP)-treated mouse model of Parkinson’s disease (PD). In mice, subchronic MPTP administration causes oxidative stress-induced apoptosis in the dopaminergic neurons of the substantia nigra pars compacta (SNpc), resulting in motor deficits. NE52-QQ57, a selective GPR4 antagonist, reduced dopaminergic neuronal loss in MPTP-treated mice, improving motor and memory functions. MPTP and NE52-QQ57 co-treatment in mice significantly decreased pro-apoptotic marker Bax protein levels and increased anti-apoptotic marker Bcl-2 protein levels in the SNpc and striatum. MPTP-induced caspase 3 activation and poly (ADP-ribose) polymerase (PARP) cleavage significantly decreased in the SNpc and striatum of mice co-treated with NE52-QQ57. MPTP and NE52-QQ57 co-treatment significantly increased tyrosine hydroxylase (TH)-positive cell numbers in the SNpc and striatum compared with MPTP alone. NE52-QQ57 and MPTP co-treatment improved rotarod and pole test–assessed motor performance and improved Y-maze test–assessed spatial memory. Our findings suggest GPR4 may represent a potential therapeutic target for PD, and GPR4 activation is involved in caspase-mediated neuronal apoptosis in the SNpc and striatum of MPTP-treated mice.

## 1. Introduction

Parkinson’s disease (PD) is a serious movement disorder and represents the second-most common progressive neurodegenerative disorder affecting individuals older than 60 years [1]. The most prominent clinical manifestations of PD include the loss of movement coordination, including the presentation of bradykinesia, asymmetric resting tremor, and rigidity [2]. The disease is typically attributed to the pathophysiologic loss or degradation of dopaminergic neurons in the substantia nigra pars compacta (SNpc) region of the brain [3]. An array of evidence suggests that the major cause of dopaminergic neuronal loss in the SNpc is reactive oxygen species (ROS)-induced oxidative stress [4]. A number of sources suggest that mitochondrial dysfunction can lead to the exacerbation of ROS generation and increased susceptibility to oxidative stress-mediated dopaminergic cell death [5].

A dopaminergic pyridine neurotoxin, 1-methyl-4-phenyl-1,2,3,6-tetrahydropyridine (MPTP), has been widely used to mimic PD in animal models. MPTP is the gold standard for toxin-based PD animal model, producing nearly all of the pathological hallmarks observed in humans with PD. When injected into specific strains of mice, such as C57BL/6, MPTP induces behavioral and systemic symptoms that resemble those presented by PD patients, such as severe motor deficits, including bradykinesia, resting tremors, rigidity, and postural instability, and dopaminergic cell death in the SNpc and striatum due to oxidative stress-mediated apoptosis [6,7]. In the brain, glial cells metabolize MPTP into the active form 1-methyl-4-phenylpyridinium (MPP^+^). MPP^+^ is transported into dopaminergic neurons through solute carrier family 6, member 3/dopamine transporter (SLC6A3/DAT) and disrupts the respiratory enzymes in mitochondria, causing oxidative damage [8,9]. MPP^+^ has been reported to result in a 5–7-fold increase in ROS generation compared with physiological levels. MPP^+^ selectively inhibits mitochondrial complex I and initiates the classical mitochondrial apoptotic pathway through a series of sequential event. The opening of the mitochondrial permeability transition pore causes the collapse of the mitochondrial membrane potential (ΔΨm), resulting in an imbalance in the pro-apoptotic Bax/Bcl-2 ratio, followed by the release of cytochrome c, the activation of caspase 3, and the proteolytic degradation of poly (ADP-ribose) polymerase (PARP), leading to the subsequent apoptosis of neuronal cells [7,8,9].

G protein-coupled receptor (GPCR) 4 (GPR4) is a proton-sensing receptor that belongs to a small family of proton-sensing GPCRs that includes ovarian cancer GPCR 1 (OGR1), G2A, and T-cell death-associated gene 8 (TDAG8), which are also known as GPR68, GPR132, and GPR65, respectively. GPR4 is a Gs, G_13_ and Gq/_11_ coupled receptor, which also modulates signals through cAMP. In several studies, GPR4 has been found to be activated within a range of physiological pH values (7.0–7.4), with little increase in cAMP observed under slightly acid conditions [10]. In the brainstem, the pH is tightly balanced and maintained between 7.0 and 7.6. In our previous studies, we demonstrated that GPR4 is active at physiological pH (approximately 7.4) in dopaminergic neuronal cells. Moreover, Hosford et al. (2018) reported that GPR4 is expressed by multiple neuronal populations and in the endothelium of both mice and rats and suggested that the pH sensitivity of GPR4 is affected by the level of expression in specific cell types [11]. A transcriptome study showed that GPR4 is highly expressed in peripheral endothelial cells (blood vessel formation) [12] and kidney (control of acid-base balance) [13]. GPR4 expression has been reported in various regions of the brainstem, particularly the retrotrapezoid nucleus (RTN), C1 catecholaminergic neurons, and serotonergic neurons of the raphe nucleus, which suggested that GPR4 may be involved in the central chemosensitivity of the RTN to CO_2_ [14]. In addition, GPR4 plays critical roles in various biological functions, such as inflammation, proliferation, paracellular gap formation between endothelial cells, apoptosis, cancer cell growth and angiogenesis [15,16,17]. Previously, the pharmacological inhibition of GPR4 was reported to remediate myocardial infarction [18], and the genetic deletion of GPR4 improved cardiac function by lowering blood pressure [19] and inhibited apoptosis, which reduced renal ischemia reperfusion injury [20] and intestinal inflammation [21]. However, no study has examined the role played by GPR4 in the apoptotic neuronal cell death associated with neurodegenerative disease. We were the first to investigate the effects of GPR4 knockout and overexpression in dopaminergic neuronal cells [22]. Our previous study showed that GPR4 knockout or pharmacological inhibition reduced neurotoxin-induced caspase 3-dependent apoptotic cell death. In this study, we used the administration of a subchronic MPTP dose to induce Parkinsonism in C57BL/6J mice to further explore the role played by GPR4 in the induction of classical mitochondrial apoptotic cell death. We used an orally active GPR4-selective antagonist to evaluate the protective effects of pharmacologically mediated GPR4 inhibition against neuronal cell loss in the SNpc, which was associated with an increase in the tyrosine hydroxylase (TH) level and the attenuation of caspase-mediated apoptotic cell death.

## 2. Results

### 2.1. Expression of GPR4 Is Upregulated in an MPTP-Induced Mouse Model of PD

**Figure 1:**
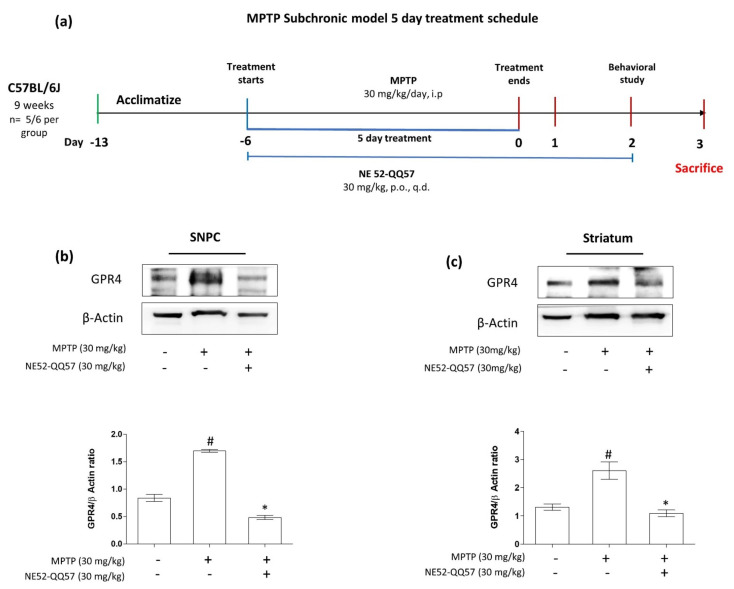
The effects of subchronic MPTP administration on the GPR4 protein expression levels in the substantia nigra and striatum of the mouse brain. (**a**) Schematic representation of the treatment and behavioral study schedule for animal experiments. GPR4 protein expression and its densitometric analysis in (**b**) the substantia nigra pars compacta (SNpc) and (**c**) the striatum measured in tissue samples collected 3 days after the final MPTP administration. β-Actin expression levels used as an internal control. Data are presented as the mean ± SEM. One-way ANOVA, followed by Tukey’s multiple comparison test, was used to compare differences between groups. # *p* < 0.05 when the MPTP-treated group was compared with the vehicle-only group; * *p* < 0.05 when other treated groups were compared with the MPTP-treated group. p.o.: oral administration; i.p.: intraperitoneal; q.d.: once a day.

The dose of NE52-QQ57 (30 mg/kg, per os (p.o.)) was adopted from the study by Hosford et al. (2018), and the MPTP (30 mg/kg, intraperitoneal (i.p.)) dose was adopted from the study by Kim et al. (2015) [11,23]. Mice were treated with MPTP alone or co-treated with NE52-QQ57 and MPTP for 5 days; in co-treated mice, NE52-QQ57 treatments were continued until the day before sacrifice. After the final MPTP injection, behavioral studies were performed, and the mice were sacrificed 3 days after the final MPTP injection to collect tissue from the substantia nigra and striatum (Figure 1a).

The activation of GPR4 is known to differ depending on the cell types and number of GPR4 receptor expression. Different regions of the brain feature very distinct cell types with different levels of GPR4 activities [11]. We investigated the expression of GPR4 in different regions of the mouse brain using immunoblot analysis (Supplementary Figure S1). We found that the GPR4 expression levels in the SNpc and striatum after subchronic MPTP administration (30 mg/kg, i.p.) for 5 days and measured the GPR4 protein expression level at the day 1, 3 and 7 after the final MPTP injection (Supplementary Figure S1). The expression level of GPR4 protein was highest in the third day.

The subchronic administration of MPTP significantly (# *p* < 0.05) increased GPR4 protein expression levels by ((1.69 ± 0.2)-fold that of vehicle-only group) in the SNpc (Figure 1b). By contrast, NE52-QQ57 co-treatment significantly (* *p* < 0.05) decreased the GPR4 protein level to ((0.48 ± 0.04)-fold that of vehicle-only group) in the SNpc (Figure 1b). In the striatum, GPR4 protein levels significantly (# *p* < 0.05) increased in the MPTP-treated group to ((2.60 ± 0.35)-fold that in the vehicle-only group) (Figure 1c). By contrast, NE52-QQ57-MPTP co-treatment significantly (* *p* < 0.05) decreased the GPR4 protein level to only ((1.09 ± 0.14)-fold that of the vehicle-only group) (Figure 1c). These data suggested that MPTP administration affected the expression level of GPR4 in the SNpc and striatum of a mice model of PD.

### 2.2. Inhibition of GPR4 Protects Against the MPTP-Induced Depletion of TH in the SNpc and Striatum

**Figure 2:**
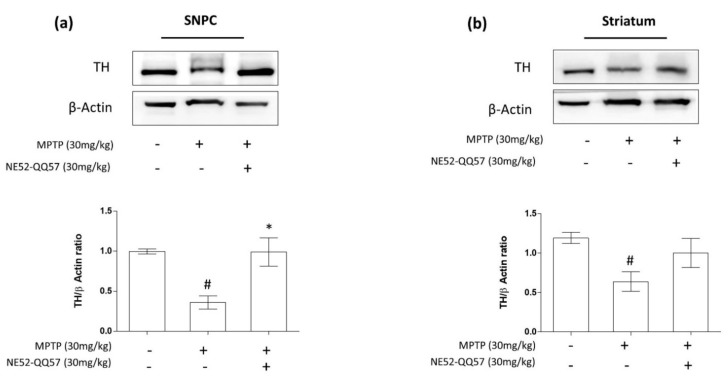
The effects of the GPR4 antagonist NE52-QQ57 on TH protein expression levels in MPTP-treated mice. MPTP was administered (30 mg/kg/day) for 5 days. Mice were sacrificed, and the substantia nigra and striatum tissues were collected 3 days after the final MPTP administration. (**a**) TH protein expression in the SNpc region of the mouse brain (*n* = 3) and densitometric analysis. (**b**) TH protein expression in the striatum region of the mouse brain (*n* = 3) and densitometric analysis. β-Actin was utilized as an internal control. Data represented the mean ± SEM. One-way ANOVA, followed by Tukey’s multiple comparison test, was used to compare differences between groups. # *p* < 0.05 when the MPTP-treated group was compared with the vehicle-only group; * *p* < 0.05 when other treated groups were compared with the MPTP-treated group.

Treatment with MPTP for 5 days significantly (# *p* < 0.05) reduced TH-immunopositivity in the SNpc to ((0.36 ± 0.09)-fold that of the vehicle-only group (Figure 2a). By contrast, NE52-QQ57 and MPTP co-treatment significantly (* *p* < 0.05) prevented TH depletion to ((0.98 ± 0.2)-fold that of the vehicle-only group) in the SNpc (Figure 2a). In the striatum, MPTP treatment for 5 days significantly (# *p* < 0.05) reduced TH-immunopositivity to (0.64 ± 0.14)-fold that of the vehicle-only group. By contrast, NE52-QQ57 and MPTP co-treatment treatment significantly (* *p* < 0.05) prevented TH depletion in the striatum, which measured (1.0 ± 0.21)-fold that of the vehicle-only group (Figure 2b). These data suggested that the depletion of TH protein by the subchronic administration of MPTP can be prevented by the inhibition of GPR4.

### 2.3. Inhibition of GPR4 Decreases MPTP-Induced Increase in the Pro-Apoptotic Bax/Bcl-2 Ratio in the SNpc and Striatum

The Bcl-2 family proteins Bax and Bcl-2 play roles as central regulators of the mitochondrial apoptotic pathway. Bcl-2 protein activity is dependent on counteracting twin Bax. The activation of Bax serves as an apoptosis initiation step, whereas Bcl-2 activation protects against programmed cell death and promotes cell survival. The activation of Bax initiates the release of cytochrome C from the mitochondrial intermembrane space to the cytosol, which activates the proteolytic caspases [24]. To investigate the effects of GPR4 inhibition on Bax and Bcl-2 protein expression, mice were co-treated with NE52-QQ57 and MPTP for 5 days, and NE52-QQ57 treatment continued until the day before sacrifice. Three days after the final MPTP injection, the SNpc and striatum were collected and processed for immunoblotting.

**Figure 3:**
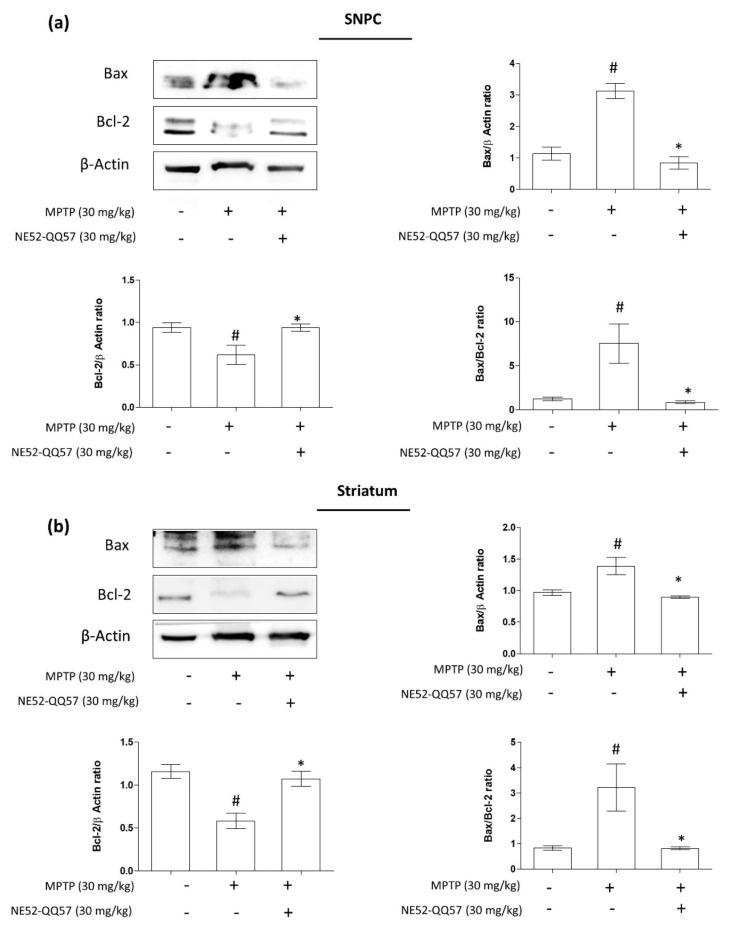
The effects of the GPR4 antagonist NE52-QQ57 on pro-apoptotic Bax and anti-apoptotic Bcl-2 protein expressions in MPTP-treated mice. MPTP was administered (30 mg/kg/day) for 5 days. Mice were sacrificed 3 days after the final MPTP injection, and the substantia nigra and striatum tissues were collected. (**a**) Bax and Bcl-2 protein expression in the SNpc region of the mouse brain (*n* = 3) and densitometric analysis. (**b**) Bax and Bcl-2 protein expression in the striatum region of the mouse brain (*n* = 3) and densitometric analysis. β-Actin was utilized as an internal control. Data are presented as the mean ± SEM. One-way ANOVA, followed by Tukey’s multiple comparison test, was used. # *p* < 0.05 when the MPTP-treated group was compared with the vehicle-only group; * *p* < 0.05 when other treated groups were compared with the MPTP-treated group.

A significant (# *p* < 0.05) (3.13 ± 0.28)-fold increase in Bax protein expression was observed in the SNpc of mice treated with MPTP compared with the level observed in vehicle-only group, whereas Bcl-2 protein expression was significantly (# *p* < 0.05) reduced to (0.57 ± 0.15)-fold that in vehicle-only group. The Bax/Bcl-2 ratio increased by more than 7-fold (7.55 ± 2.57) that of the vehicle-only group (Figure 3a). Additionally, co-treatment with NE52-QQ57 and MPTP significantly (* *p* < 0.05) prevented the increase in Bax expression, which was (0.84 ± 0.22)-fold that of the vehicle-only group, and the decrease in Bcl-2 expression ((0.95 ± 0.05)-fold that of the vehicle-only group) in the SNpc compared with the effects in group treated with MPTP alone (Figure 3a). The Bax/Bcl-2 ratio in the SNpc of the NE52-QQ57 and MPTP co-treated group was also significantly (* *p* < 0.05) lower ((0.86 ± 0.17)-fold that of the vehicle-only group) than that in the MPTP-treated group.

In the tissue collected from the striatum of MPTP-treated mice, the expression level of Bax was significantly (# *p* < 0.05) increased to (1.39 ± 0.16)-fold and the level of Bcl-2 expression was (0.52 ± 0.09)-fold that of the vehicle-only group. The Bax/Bcl-2 ratio was significantly (# *p* < 0.05) increased in MPTP-treat mice, at (3.22 ± 0.82)-fold that of the vehicle-only group (Figure 3b). However, in the NE52-QQ57 and MPTP co-treated group, a significant (* *p* < 0.05) decrease in Bax expression was observed ((0.89 ± 0.024)-fold that of the vehicle-only group) and less depletion of Bcl-2 protein expression was observed ((1.11 ± 0.11)-fold that of the vehicle-only group) compared with the MPTP-treated group. Moreover, the Bax/Bcl-2 ratio in the NE52-QQ57 and MPTP co-treated group was also significantly (* *p* < 0.05) lower than that in the MPTP-treated group ((0.82 ± 0.06)-fold that of the vehicle-only group) (Figure 3a). These data suggested that the subchronic administration of MPTP caused an imbalance in the mitochondrial Bax/Bcl-2 protein ratio, which might initiate the mitochondrial caspase-mediated apoptotic pathway. Treatment with the selective GPR4 antagonist NE52-QQ57 prevented the increase in pro-apoptotic Bax protein expression and prevented the depletion of anti-apoptotic Bcl-2 protein expression, indicating the anti-apoptotic effects of NE52-QQ57.

### 2.4. Effects of GPR4 Inhibition on PARP Cleavage and Caspase 3 Activity in the SNpc and Striatum of MPTP-Induced PD Model Mice

In response to oxidative stress, PARP becomes the most abundant nuclear enzyme. PARP cleavage, a reliable marker of apoptosis, is performed by the DEVD-ase caspases, a family of proteases, such as caspase 3, which are activated during apoptosis [25,26]. To further assess the effects of GPR4 inhibition, mediated by NE52-QQ57, against apoptosis in dopaminergic neurons, we measured the levels of cleaved PARP, its proteolytic enzyme caspase 3, and caspase activity in the SNpc and striatum tissue collected from MPTP-treated mice.

**Figure 4:**
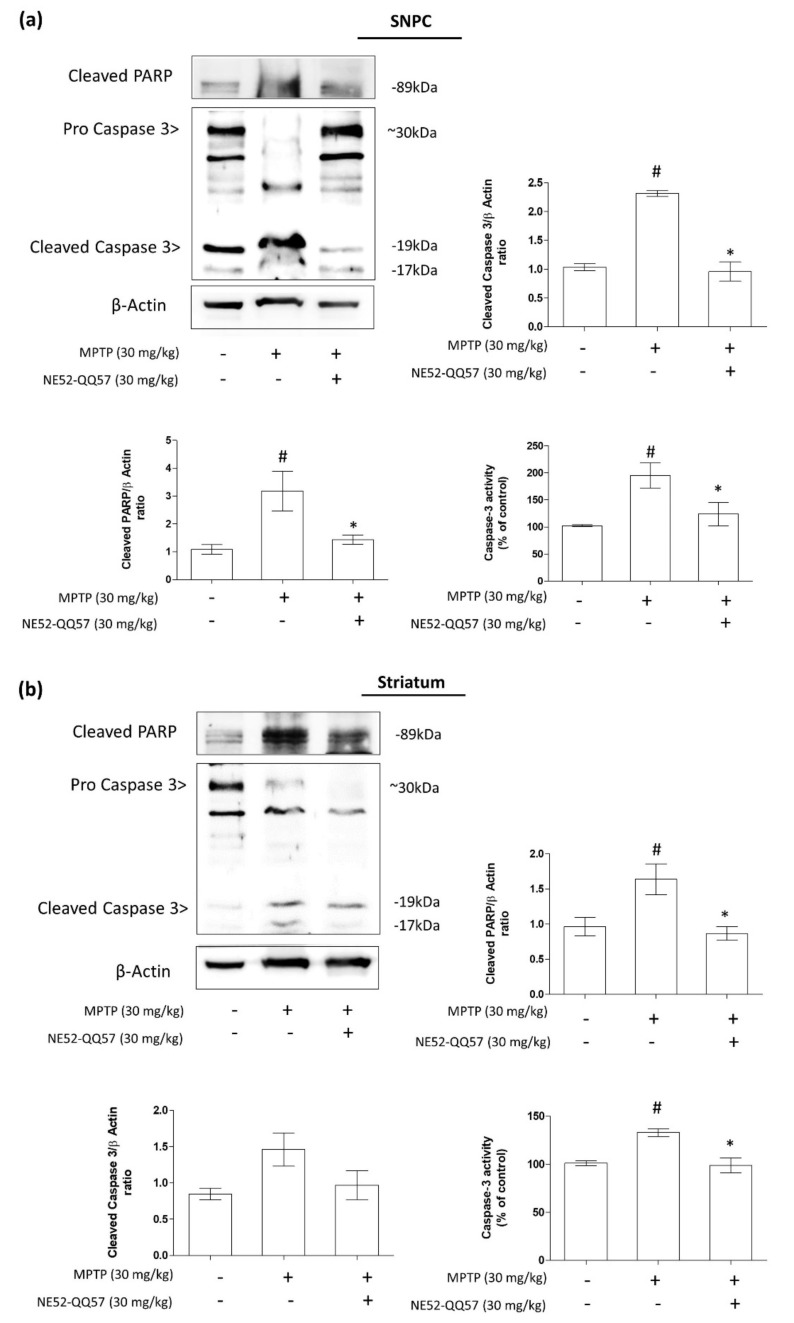
The effects of the GPR4 antagonist NE52-QQ57 on the expression of pro-apoptotic proteins, cleaved PARP and cleaved caspase 3, and caspase 3 activity in MPTP-treated mice. MPTP was administered (30 mg/kg/day) for 5 days. Mice were sacrificed, and the substantia nigra and striatum tissue was collected 3 days after the final MPTP administration. (**a**) Cleaved PARP-1 and cleaved caspase 3 protein expression and caspase 3 activity assay in the mouse SNpc (*n* = 3) and densitometric analysis. (**b**) Cleaved PARP-1 and cleaved caspase 3 protein expression and caspase 3 activity assay in the mouse striatum (*n* = 3) and densitometric analysis. β-Actin was utilized as an internal control. Data are presented as the mean ± SEM. One-way ANOVA, followed by Tukey’s multiple comparison test, was used to compare differences between groups. # *p* < 0.05 when the MPTP-treated group was compared with the vehicle-only group; * *p* < 0.05 when the other treated groups were compared with the MPTP-treated group.

After treatment with MPTP for 5 days, the level of cleaved PARP protein expression in the mouse SNpc increased significantly (# *p* < 0.05) to (3.78 ± 0.6)-fold that in vehicle-only group, whereas cleaved caspase 3 protein increased to (2.32 ± 0.06)-fold that of the vehicle-only group (# *p* < 0.05) (Figure 4a). Additionally, in a separate quantitative caspase 3 activity assay, the MPTP-treated group showed nearly double the caspase 3 activity (194.923% ± 25.86%) as that in the vehicle-only group. By contrast, co-treatment with NE52-QQ57 and MPTP reduced PARP cleavage ((1.56 ± 0.14)-fold that of the vehicle-only group), reduced cleaved caspase 3 ((0.96 ± 0.19)-fold that of the vehicle-only group), and prevented the increase in caspase 3 activity (123.847% ± 23.63%) compared with the group treated with MPTP alone.

However, in the striatum, the MPTP-treated mice presented with significantly (# *p* < 0.05) increased levels of cleaved PARP to (1.84 ± 0.11)-fold that of the vehicle-only group and increased cleaved caspase 3 to (1.46 ± 0.26)-fold that of the vehicle-only group. The quantitative analysis of caspase 3 activity showed a significant (# *p* < 0.05) increase (133.03% ± 4.87%) in caspase 3 activity in MPTP-treated mice relative to the vehicle-only group, whereas co-treatment with NE52-QQ57 and MPTP prevented the increase in cleaved PARP((0.85 ± 0.14)-fold that of the vehicle-only group) and cleaved caspase 3 ((0.97 ± 0.23)-fold that of the vehicle-only group), and significantly (* *p* < 0.05) prevented the increase in caspase 3 activity (98.87% ± 9.02%) (Figure 4b). Overall, these data suggested that the inhibition of GPR4 had a strong effect on the inhibition of apoptotic cell death through the inhibition of mitochondrial caspase 3-mediated PARP cleavage. However, although GPR4 inhibition prevented the MPTP-mediated increase in caspase 3 activity in both the SNpc and striatum tissues, the protective effect of NE52-QQ57 was higher in the SNpc than in the striatum.

### 2.5. Effect of GPR4 Antagonist on the MPTP-Induced Degradation of TH-Positive Cells in SNpc and Striatum

TH is an enzyme that converts L-tyrosine to L-3,4-dihydroxyphenylalanine (L-DOPA). The conversion from L-tyrosine to L-DOPA is the rate-limiting and initial step in the dopamine biosynthesis pathway in dopaminergic neurons [27]; therefore, TH is widely used as a marker for dopaminergic neurons in the central nervous system. To evaluate the effects of NE52-QQ57 treatment on TH-positive cells in the SNpc and striatum, mice were treated with MPTP, either alone or with NE52-QQ57, for 5 days. NE52-QQ57 treatment was continued until the day before sacrifice. Three days after the final MPTP injection, the mice were deeply anaesthetized, followed by transcardial transfusion and fixation, and the brains were isolated. Immunohistochemistry was performed to measure the immunoreactivity of TH-positive neurons in sections of the SNpc and striatum.

**Figure 5:**
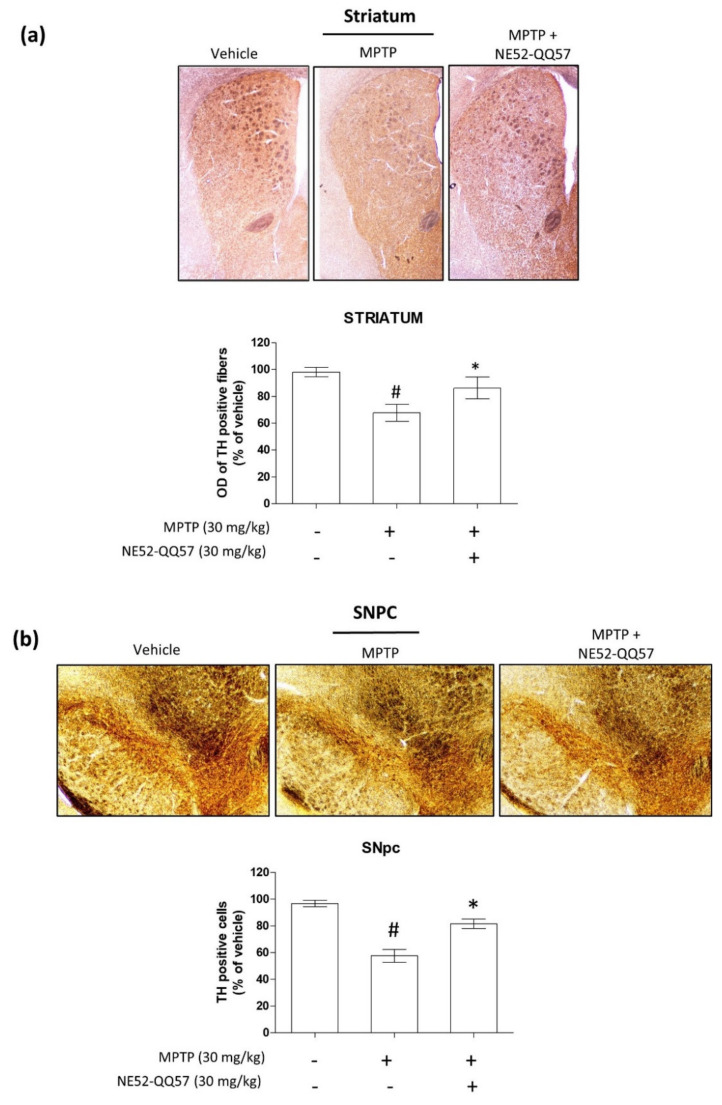
GPR4 inhibition attenuates the depletion of TH-positive cells in the striatum and SNpc of a subchronic MPTP-treated mouse model of PD. MPTP was administered (30 mg/kg/day) for 5 days. After performing behavioral experiments, the mice were anaesthetized for the immunohistochemical study three days after the final MPTP administration. Mice were sacrificed, and the whole brain was isolated to collect sections from the SNpc and striatum regions. (**a**). Representative images of TH-positive fibers immunoreactivity (IR) in sections of the striatum, with densitometric analysis of optical density of TH positive fibers (*n* = 3–4). (**b**). Representative images of TH-positive cell IR in the SNpc sections, with densitometric analysis for TH positive cells (*n* = 3–4). Data are presented as mean ± SEM. One-way ANOVA, followed by Tukey’s multiple comparison test, was used to determine significant differences between groups. # *p* < 0.05 when the MPTP-treated group was compared with the vehicle-only group; * *p* < 0.05 when the other treated groups were compared with the MPTP-treated group.

In the striatum, a significant reduction in TH-positive cells was observed after the subchronic administration of MPTP. By contrast, the co-treatment with NE52-QQ57 protected against the loss of TH-positive cells (Figure 5a). Densitometric analyses of the images showed that MPTP treatment significantly (# *p* < 0.05) reduced TH-positive cells (87.07% ± 1.53%) in comparison with the vehicle-only group. NE52-QQ57 AND mptpco-treatment significantly (* *p* < 0.05) prevented the loss of TH-positive cells (103.73% ± 2.56%).

In the SNpc tissues, a similar phenomenon was observed, with MPTP significantly reducing the number of TH-positive cells and NE52-QQ57 and MPTP co-treatment resulting in the reduced depletion of TH-positive cells (Figure 5b). Quantitative evaluations of the images showed the significant depletion of TH-positive cells (62.46% ± 6.56%) compared with the vehicle-only group. In the MPTP and NE52-QQ57 co-treated group, significant (* *p* < 0.05) protection against the depletion of TH-positive cells (101.74% ± 6.53%) was observed. Although both the SNpc and striatum incurred significant MPTP-induced damage to TH-positive dopaminergic neurons, the degree of depletion in the SNpc was higher than that in the striatum. Overall, these data suggested that NE52-QQ57 strongly protected against the loss of TH-positive cells in both major locations associated with TH-positive cell depletion in the brains of MPTP-treated mice.

### 2.6. Effect of GPR4 Antagonist on the Motor Coordination Function (Rotarod), Bradykinesia (Pole Test) and Spontaneous Alternation Performance (Y-maze Test) on MPTP-Induced PD Mouse Model

**Figure 6:**
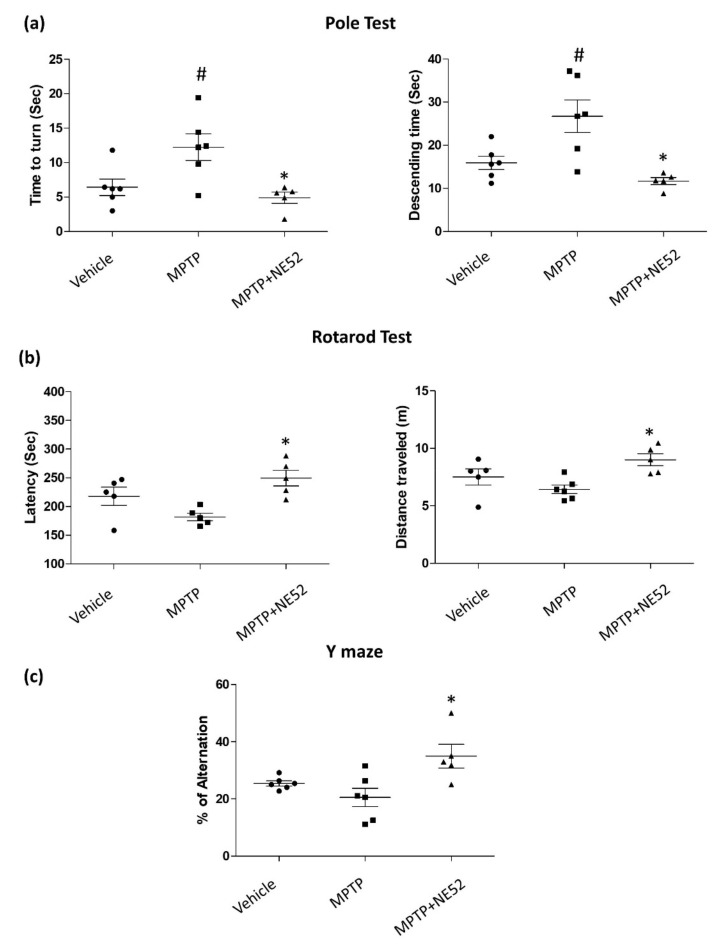
GPR4 inhibition attenuates motor deficits in a subchronic MPTP-treated mouse model of PD. MPTP was administered (30 mg/kg/day) for 5 days. Behavioral experiments were performed three days after the final MPTP administration (**a**) PD-like bradykinesia was measured using a pole test. (**b**) Motor coordination function was measured using a Rotarod test. Both the fall latency and the distance travelled were measured. (**c**) Spatial memory measured using Y-maze. Each dot represents the average of five individual trials, and the thick bar represents the mean ± standard deviation. Data represented as mean ± SEM (*n* = 5 of triplicates). One-way ANOVA, followed by Tukey’s multiple comparison test, was used to compare differences between groups. # *p* < 0.05 when the MPTP-treated group is compared with the vehicle-only group; * *p* < 0.05 other treated groups compared with the MPTP-treated group.

To investigate the effect of GPR4 inhibition on MPTP-induced behavioral deficits, we performed the pole test, rotarod test, and Y-maze test to evaluate motor coordination function, bradykinesia, and spatial memory performance. Three days after the final MPTP treatment, all behavioral tests were performed. In the pole test, the MPTP-treated mice group showed a significant increase (# *p* < 0.05) in the time to turn (12.24 ± 1.92 s) and the descending time (26.72 ± 3.75 s) compared with the vehicle-only group (6.44 ± 1.19 s). The latency to fall (188.62 ± 7.87 s) and distance travelled (6.434 ± 0.34 m) in the rotarod test also decreased in the MPTP-treated mouse group compared with those in the vehicle-only group (217.85 ± 12.867 s) (7.51 ± 0.57 m). Additionally, MPTP treatment decreased the percentage of alteration (20.52 ± 3.2%) during the Y-maze test compared with the vehicle-only group (25.44% ± 089%). By contrast, the NE52-QQ57 and MPTP co-treatment group showed a significantly (* *p* < 0.05) shorter time to turn (4.9 ± 0.66 s) and descend (11.7 ± 0.65 s) on the pole test than those for the MPTP-only treatment group (Figure 6a). The NE52-QQ57 and MPTP co-treatment group also showed a significantly (* *p* < 0.05) higher fall latency (249.5 ± 11.19 s) and increased distance travelled (9.01 ± 0.43 m) compared with those in the MPTP-treated group (Figure 6b). In the Y-maze test, the percentage of alteration (32.95% ± 4.17%) was significantly (* *p* < 0.05) higher than that in the MPTP-treated group (Figure 6c). Overall, these data suggested that GPR4 plays an important role in the MPTP-induced pathogenesis of PD. Thus, the inhibition of GPR4 was able to improve the MPTP-induced behavioral deficits in a mouse model of PD.

## 3. Discussion

In this study, we investigated the effects of the pharmacological inhibition of GPR4 on the mitochondrial oxidative stress-induced apoptotic cell death in a PD mouse model. The activation of GPR4 has been reported at many different pH ranges in various cell types and animal models. Hosford and his team also reported the expression of GPR4 in the cerebrovascular endothelium and the neurons of the retro-trapezoidal nucleus of the locus coeruleus, the dorsal raphe, and the lateral septum of mice at physiological pH values. We have shown the GPR4 is activated at physiological pH values (Supplementary Data 1) and is capable of potentiating the effects of MPP^+^, an active metabolite of MPTP [22]. In this study, we aimed to determine whether MPTP treatment affected the GPR4 expression level over time in the cerebellum, cerebral cortex, substantia nigra, hippocampus, pons medulla, midbrain, or striatum of mouse brains 1, 3, and 7 days after the final MPTP injection. No significant changes in GRP4 expression were observed in the cerebellum and cerebral cortex following MPTP treatment. By contrast, significantly increased GPR4 levels were observed in the SNpc, hippocampus, midbrain, and striatum 3 days after the final MPTP administration, whereas these levels decreased slightly by 7 days after the final MPTP administration (Supplementary Figure S1). Thus, we selected 3 days after the final MPTP treatment for the collection of SNpc and striatum tissues and the performance of behavioral studies. Interestingly, in pons medulla, the GPR4 protein level gradually increased from days 3–7 following MPTP administration. As suggested by Hosford et al. (2018), GPR4 activity may be associated with the GPR4 expression level in different tissues, which may explain the variations in GPR4 expression among different populations of neuronal cells following MPTP administration.

The SNpc, hippocampus, striatum, and midbrain are the primary regions affected by PD pathology and likely represent the sites where MPTP is converted to MPP^+^ due to the high numbers of glial cells and TH-positive dopaminergic cells. Our immunoblot and immunohistochemistry (IHC) results also suggested that the GPR4 expression may be involved in the apoptotic cell death of TH-positive dopaminergic neurons in the midbrain, SNpc, and striatum 3 days after the final administration of MPTP. To investigate the effects of GPR4 inhibition on neuronal loss, memory deficits, and motor performance, we used the selective GPR4 antagonist NE52-QQ57 in an MPTP-induced mouse model of PD. TH, an essential enzyme involved in dopamine biosynthesis, converts tyrosine to dopamine using tetrahydrobiopterin and molecular oxygen [27]. Decreased TH activity is positively correlated with the loss of dopaminergic neuronal cells. Behavioral deficits are also correlated with the number of TH-positive neurons in the SNpc and striatum [23]. We found that GPR4 inhibition protected against the loss of TH-positive neurons in the striatum and SNpc following MPTP treatment, which suggested that GPR4 inhibition may protect against the dopaminergic neuron loss associated with disruptions in motor activity and memory deficits.

To further investigate the mechanism through which selective GPR4 inhibition prevents MPTP-induced neuronal loss, we examined the effects of GPR4 inhibition on the mitochondrial oxidative stress-mediated apoptotic pathway. We investigated the ratio between Bax and Bcl-2 as an early indicator of apoptotic cascade activation [28,29]. MPTP-induced apoptotic cell death is characterized by the hallmarks of an increase in the Bax/Bcl-2 ratio, the release of cytochrome C, and caspase 3 activation, which cleaves PARP and induces apoptotic cell death [30]. Previous studies have suggested that MPTP administration upregulates the expression of Bax and decreases the expression level of Bcl-2 in the SNpc and striatum of mice. These changes were found to parallel MPTP-induced dopaminergic neurodegeneration. Studies have also shown that mice lacking Bax are resistant to MPTP-induced neuronal damage [30,31]. In our study, GPR4 inhibition significantly prevented the increase in Bax expression and the decrease in Bcl-2 expression, restoring the Bax/Bcl-2 ratio in the SNpc and striatum of MPTP-treated mice. Although the changes in the Bax/Bcl-2 ratio were more pronounced in the SNpc than in the striatum, the level of restoration induced by NE52-QQ57 treatment was almost the same between these two regions. Many studies have suggested that the balance between Bax and Bcl-2 is indicative of protection against apoptotic cell death [32,33,34]. MPTP-mediated mitochondrial oxidative stress activation causes a series of downstream events, including caspase activation and the cleavage of PARP, which consumes ATP and acutely depletes the cellular energy stores of dopaminergic neurons. We further investigated the cleavage of caspase 3, caspase 3 activity, and PARP cleavage. PARP is a major substrate of caspase activity and represents a valuable apoptosis marker. MPTP-induced mitochondrial oxidative stress induces a dramatic increase in caspase 3 activity and PARP cleavage associated with neuronal cell death, and caspase 3 has been reported to act as the final effector of caspase-dependent apoptosis [35,36]. Our results showed that MPTP dramatically increased the protein levels of both cleaved caspase 3 and cleaved PARP in the SNpc and striatum. Additionally, caspase 3 activity was significantly increased in both the SNpc and striatum. Although the fold-change in cleaved caspase 3 and PARP protein levels and caspase 3 activity was not the same in these two regions, co-treatment with NE52-QQ57 significantly prevented the cleavage of both proteins and inhibited caspase3 activity in both brain areas. These findings demonstrate that the pharmacological inhibition of GPR4 prevented MPTP-induced mitochondrial oxidative stress and prevented the initiation of the caspase 3-dependent apoptotic cell death pathway. This result aligns with our previously published in vitro data that examined both the genetic and pharmacological inhibition of GPR4 in a human dopaminergic cell line [22].

To further confirm the degree of dopaminergic cell loss prevented by GPR4 inhibition, we investigated the immunoreactivity of TH-positive cells in SNpc and striatum tissues using IHC. Surprisingly, the IHC results for the SNpc and striatum sections of the brain showed that GPR4 inhibition significantly prevented the depletion of TH-positive cells in MPTP-treated mice. This finding supports our immunoblot data showing the effects of MPTP and NE52-QQ57 co-treatment on TH protein levels in the SNpc and striatum. The depletion of TH-positive cells in MPTP-treated mice in this study was similar to the level of depletion previously reported for a subchronic MPTP-induced model of PD [37]. Our findings indicated similarity with previous studies, demonstrating that TH activity correlates with behavioral deficits in a toxin-induced animal model of PD [23,28].

We investigated whether the pharmacological inhibition of GPR4 had any effects on memory impairments and behavioral deficits by performing rotarod, Y-maze, and pole tests. The pole test is used as a behavioral test to assess bradykinesia in PD mouse models [38]. The pharmacological inhibition of GPR4 improved MPTP-induced bradykinesia in mice. To evaluate motor activity, we used the rotarod test, which is commonly used to assess mouse models of PD [39]. The rotarod results showed that the GPR4 antagonist significantly prevented MPTP-induced motor deficits in mice. Cognitive impairments associated with spatial memory have been reported after the subchronic administration of MPTP in various studies [40,41,42]. To assess the impairment of spatial memory, we used the Y-maze test, which is widely used to assess MPTP-treated subchronic models of PD. Our study showed that the inhibition of GPR4 activity significantly improved the impaired spatial memory of MPTP-treated mice. However, in the rotarod test, neither the fall latency nor the distance travelled was significantly different between groups, and no significant difference was observed for the Y-maze test. However, the fall latency observed in this study was similar to the fall latency reported by Liu et al. (2018), which indicated that the PD mouse model was successfully established [43]. Therefore, the lack of statistical significance could be attributed to the number of mice used in the study. Interestingly, the immunoblot data also revealed GPR4 expression in the hippocampus region, suggesting that GPR4 activity may be associated with the memory deficits that manifest in neurodegenerative disorders, including PD. These findings provide a basis for performing further investigations into the involvement of GPR4 in memory deficits.

Taken together, the results of our study suggested that the subchronic administration of MPTP in mice induced classical mitochondrial oxidative stress-mediated dopaminergic neuronal loss in the SNpc and striatum. MPTP-induced mitochondrial oxidative stress reduces the Bax/Bcl-2 ratio, increases the caspase 3 activity-mediated proteolytic degradation of PARP, and triggers subsequent apoptosis. Thus, the number of TH-positive neurons becomes depleted from the SNpc and striatum over time. As a result, mice develop motor deficits and cognitive impairments, which can be restored by the pharmacological inhibition of GPR4. In our previous study, we speculated that GPR4 activation potentiates the neurotoxin-mediated apoptotic cell death through the phospholipase C-beta (PLC-β)-mediated release of intracellular calcium. We were unable to investigate this phenomenon in an animal model of PD. However, this study encourages further investigations into the cell-specific expression of GPR4 in the SNpc and striatum to better understand howGPR4 inhibition ameliorates the incidence of apoptotic cell death in PD.

## 4. Materials and Methods

### 4.1. Reagents and Antibodies

NE 52QQ57 was purchased from MedChemExpress USA (Monmouth Junction, NJ, USA). 3-(3,4-dimehylthiazol-2-yl)-2,5-diphenyl-tetrazolium bromide (MTT) was obtained from Sigma-Aldrich (St. Louis, MO, USA). Radioimmunoprecipitation assay (RIPA) buffer (10×) was purchased from Millipore (Milford, MA, USA). Tween 80 was purchased from Calbiochem (Gibbstown, NJ, USA). All other chemicals used in this research were of analytical grade and were purchased from Sigma-Aldrich unless otherwise noted.

### 4.2. Animals and MPTP and NE 52QQ57 Administration

Male C57BL/6J mice (age: 8–9 weeks; weight: 25–28 g; Samtako Bio Korea, Gyeonggi-do, Korea) were acclimatized for 14 days prior to drug treatment. The Institutional Animal Care and Use Committee of Konkuk University approved all the animal experiments and the experimental procedures (KU20207, 22 December 2020). The animals were housed in a controlled environment (23 ± 1 °C and 50% ± 5% humidity) and were allowed access to food and water ad libitum. The room lights were on between 8:00 a.m. and 8:00 p.m. For the selective inhibition of GPR4, the orally active GPR4 antagonist NE52-QQ57 was used. The dose of NE52-QQ57 was adjusted based on the previously published article by Hosford et al. [11].

Seventy-two mice were randomly divided into the following nine groups, comprised of 8 animals each: Vehicle group, MPTP (30 mg/kg/10 mL) group, and NE52-QQ57 (30 mg/kg/10 mL) MPTP (30 mg/kg/10 mL) group. As demonstrated in the experimental design, 30 mg/kg MPTP in a 10 mL volume was administered i.p. each day 5 for days to all MPTP-treated animals. The vehicle group was administered an equal volume of vehicle (saline). NE52-QQ57 (30 mg/kg) in a 10 mL volume was orally administered daily, 1 h before the MPTP injection, starting 1 day prior to MPTP injection until 1 day before sacrifice. The animals in each treatment group were divided into three subgroups that were sacrificed 1, 3, or 7 days after the final MPTP injection. MPTP solution was freshly prepared in saline before each use. NE52-QQ57 was suspended in 10% DMSO, 0.5% methylcellulose solution containing 0.5% Tween 80, before dosing. A plain methylcellulose solution was orally administered as the vehicle to the vehicle-only group.

### 4.3. Immunoblot Analysis

The immunoblot assays of target proteins were performed using our general lab protocol, which was reported in a previous publication [22]. First, mouse brain tissues from different sections of the brain (cerebellum, cerebral cortex, substantia nigra, hippocampus, pons medulla, midbrain, and striatum) were collected, and cell lysates were prepared using the RIPA lysis buffer (Milford, MA, USA). The protein concentration of each tissue sample was measured using a DC Protein Assay kit (Bio-Rad). Equal amounts of protein (20–30 µg) were separated using 8%, 10%, and 12% sodium dodecyl sulfate-polyacrylamide gel electrophoresis and transferred to nitrocellulose membranes (Millipore, Bedford, MA, USA). Each membrane was individually incubated overnight at 4 °C with primary antibodies against GPR4 (1:500; Novus Biologicals, Centennial, CO, USA); Bax (1:1000; Cell Signaling Co., Boston, MA, USA); BCL-2 (1:1000), caspase 3 (1:1000), cleaved caspase 3 (1:1000), and cleaved PARP (1:1000) from Santa Cruz Biotechnology (Santa Cruz, CA, USA); phosphatidylinositol 4,5-bisphosphate (PIP2, 1:500; Abcam, Cambridge, United Kingdom); and β-Actin (1:2000; Sigma-Aldrich, St. Louis, MO, USA). A 1-h incubation was performed using secondary horseradish peroxidase (HRP)-conjugated antibodies (1:2000; Cell Signaling, MA). To visualize the bands, blots were incubated with Biorad-ECL (Bio-Rad Laboratories; Hercules, CA, USA) and photographed using ImageQuant™ LAS 500 (GE Healthcare; Chicago, IL, USA). ImageJ (NIH; Bethesda, MD, USA) software was used to calculate the pixel intensity for each band. β-Actin was used to normalized the band intensity and to quantify the relative expression of other bands.

### 4.4. Assessment of Caspase-3 Activity

The caspase 3 activity in brain tissue lysates was measured using the Colorimetric Caspase-3 Assay Kit (Sigma-Aldrich, St. Louis, MO, USA), as described previously [22]. A 200 µL reaction mixture containing 50 µL of protein, was placed in each well of a 96-well plate and incubated at 37 °C for 180 min. The absorbance values of the reaction mixtures were measured at the wavelength of 405 nm in a microplate reader (Tecan Microplate Reader; Meilen, Zurich, Switzerland).

### 4.5. Immunohistochemistry

Mice were anesthetized with chloroform after performing the behavioral experiments and transcardially perfused with phosphate-buffered saline (PBS), followed by 4% paraformaldehyde (PFA) [6]. After perfusion fixation, the brains were removed and immersed in 4% PFA at 4 °C and dehydrated in 30% sucrose solution at 4 °C, followed by embedding in tissue freezing medium (Leica, Gmbh Heidelberger, Germany). Frozen brans were cut in a freezing microtome. Free-floating sections (45 µM) of the striatum and SNpc regions were used for immunohistochemistry (as described previously) [23]. The primary antibody was a rabbit anti-TH antibody (1:1000; Millipore, Darmstadt, Germany). The secondary antibody was a biotinylated anti-rabbit antibody (Vector Laboratories, Burlingame, CA, USA) incubated for 1 h, followed by incubation in the Vectastain Elite avidin-biotin-peroxidase complex (ABC) kit (Vector Laboratories, Darmstadt, Germany) for 60 min at room temperature, as described by the manufacturer’s recommendations. Finally, the sections were incubated in a 3,3′diaminobenzidine (DAB) substrate kit (Vector Laboratories) for color development, dried, and mounted on glass slides. Images of stained sections were taken by a bright-field microscope (Carl Zeiss Inc., Oberkochen, Germany). ImageJ software (NIH) is used to calculate the number of TH-positive cells in the SNpc and striatum. Data are presented as the percentage of the vehicle group values.

### 4.6. Behavioural Studies

Rotarod test: The rotarod test was performed as described previously, with slight modifications [31]. After 1, 3, and 7 days following the final MPTP injection, rotarod performance was evaluated on the suspended rod (diameter: 3 cm) of an accelerating rotarod apparatus, which accelerated at a constant rate from 1 to 40 rpm over 300 s. Mice were trained for 3 consecutive days, and they were placed on the rod for five trials. The time was automatically recorded for each trial. The distance travelled before stopping or falling was also measured. A trial was considered ended when any mouse fell off the rotarod or when the time reached 300 s. A 180 s resting interval was allowed between each trial.

Pole test: The pole test was used to investigate bradykinesia in mice and was conducted based on our previous work, with slight modifications [23]. Mice were placed at the top of a rough-surfaced wooden pole, which was 8 mm in diameter and 55 cm tall. The total time necessary for the mouse to descend the pole and the time to turn was measured. The time until the mouse reached the floor is referred to as the time to descend. The delay or extension of the time typically required to complete the test was considered to reflect bradykinesia. Each mouse was tested five times in succession.

Y-Maze test: To assess the spontaneous alternation performance, a Y-maze test was performed using a similar procedure as described previously [23]. Spontaneous alternation performances were assessed by recording the animal behavior during a single session in a Y-maze. In the Y-maze apparatus, each arm was 40 cm long, 12 cm wide, and 30 cm in height. Each Y-maze naïve mouse was placed at the end of one arm and allowed to move freely through every arm of the maze. During an 8 min session, the total entry into each arm and the spontaneous alternation of arm entries were recorded visually. An arm entry was considered to be accomplished when the hind paws of a mouse were completely placed in any arm. Alternation was considered when a mouse entered into all three arms on overlapping triplet sets. Spontaneous alternation performance was calculated as the percentage alternation based on the formula: percent alternation = ((number of alternations)/(total number of arm entries − 2)) × 100%.

### 4.7. Statistical Analyses

GraphPad Prism software version 5 (GraphPad, La Jolla, CA, USA) was used to perform statistical analyses. Data are presented as the mean ± standard error of the mean (SEM) for three to five independent experiments. One-way analysis of variance (ANOVA), followed by Tukey’s multiple comparison test, was performed to determine significant differences between the nontreated and treated groups. A *p*-value < 0.05 was considered significant.

## Data Availability

Data is contained within the article or Supplementary Materials.

## Supplementary Materials

The following are available online at https://www.mdpi.com/article/10.3390/ijms22094674/s1, Figure S1: The effect of sub chronic administration MPTP, on the GPR4 Protein expression in different regions of mice brain. and Supplementary Data 1: pH condition changes the expression level of GPR4 and the expression of apoptotic markers.

## Abbreviations

MPTP	1-Methyl-4-Phenyl-1,2,3,6-Tetrahydropyridine
MPP^+^	1-Methyl-4-Phenylpyridinium Ion
MTT	3-(3,4-Dimehylthiazol-2-Yl)-2,5-Diphenyl-Tetrazolium Bromide
ER	Endoplasmic Reticulum
HUVEC	Human Umbilical Vein Endothelial Cells
L-DOPA	L-tyrosine to L-3,4-dihydroxyphenylalanine
MMP	Mitochondrial Membrane Potential
mPTP	Mitochondrial Permeability Transition Pore
PIP2	Phosphatidylinositol Biphosphate
PARP	Poly (ADP-Ribose) Polymerase
SNpc	Substantia Nigra Pars Compacta
TH	Tyrosine hydroxylase
TDAG8	T-Cell Death-Associated Gene 8
OGR1	The Ovarian Cancer G Protein-Coupled Receptor 1
