# Supplementary material for: The Neuroprotective Effects of GPR4 Inhibition through the Attenuation of Caspase Mediated Apoptotic Cell Death in an MPTP Induced Mouse Model of Parkinson’s Disease
Source: Int J Mol Sci. 2021 Apr 28;22(9):4674. doi: 10.3390/ijms22094674 (PMC8125349; doi:10.3390/ijms22094674)
Supplement: Supplementary file 1 [file ijms-22-04674-s001.zip › ijms-1194070-supplementary/Supplementary data 1.pdf]

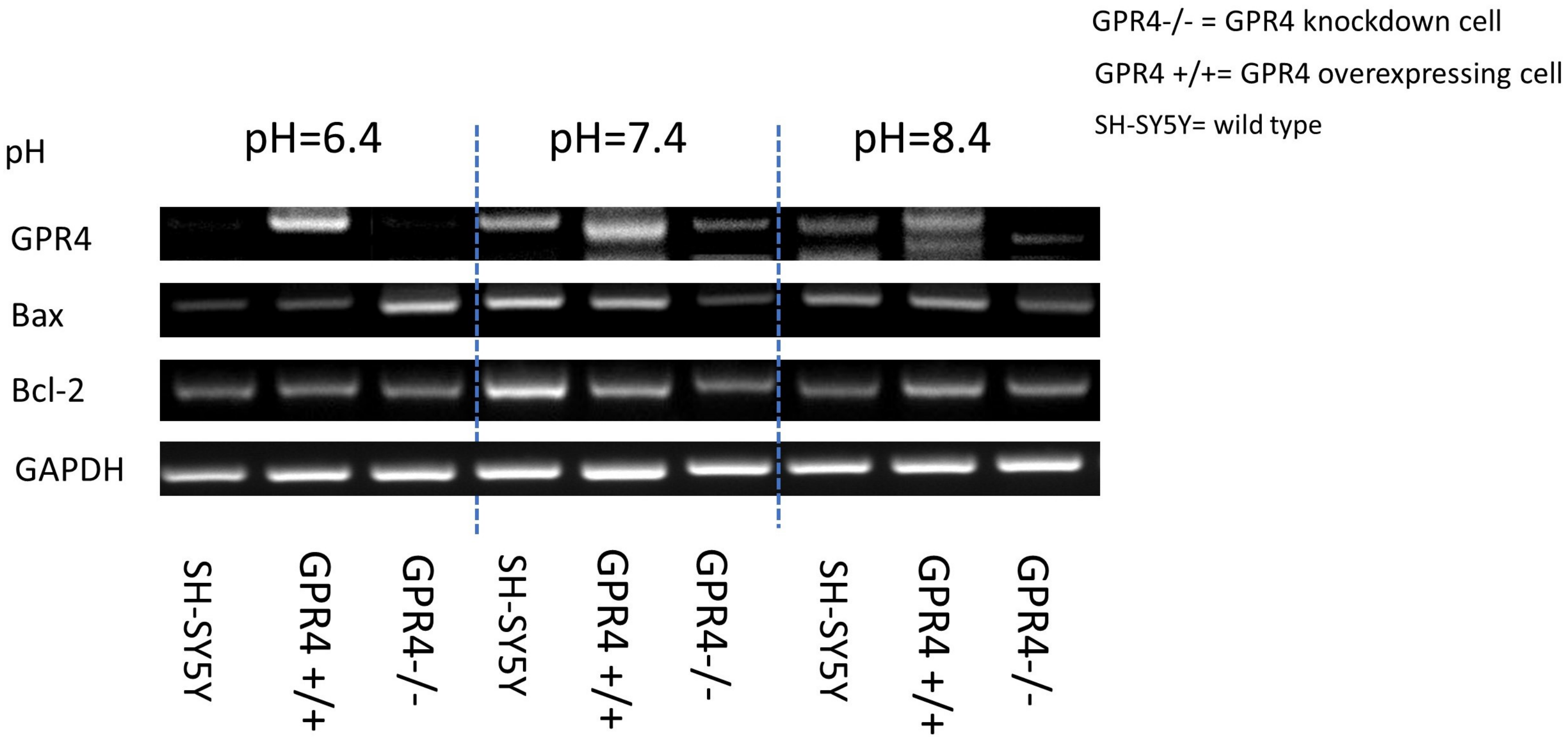

Supplementary figure 2: pH condition changes the expression level of GPR4 and the expression of apoptotic markers. mRNA expression level of GPR4 and Bax, Bcl-2 apoptotic markers in the SH-SY5Y. SH-SY5Y stably expressing GPR4 and GPR4 knocked out stable SH-SY5Y cells treated with different pH mediums for 6 hours. GAPDH was used as internal control.
