# Supplementary material for: The Neuroprotective Effects of GPR4 Inhibition through the Attenuation of Caspase Mediated Apoptotic Cell Death in an MPTP Induced Mouse Model of Parkinson’s Disease
Source: Int J Mol Sci. 2021 Apr 28;22(9):4674. doi: 10.3390/ijms22094674 (PMC8125349; doi:10.3390/ijms22094674)
Supplement: Supplementary file 1 [file ijms-22-04674-s001.zip › ijms-1194070-supplementary/Supplementary figure S1.pdf]

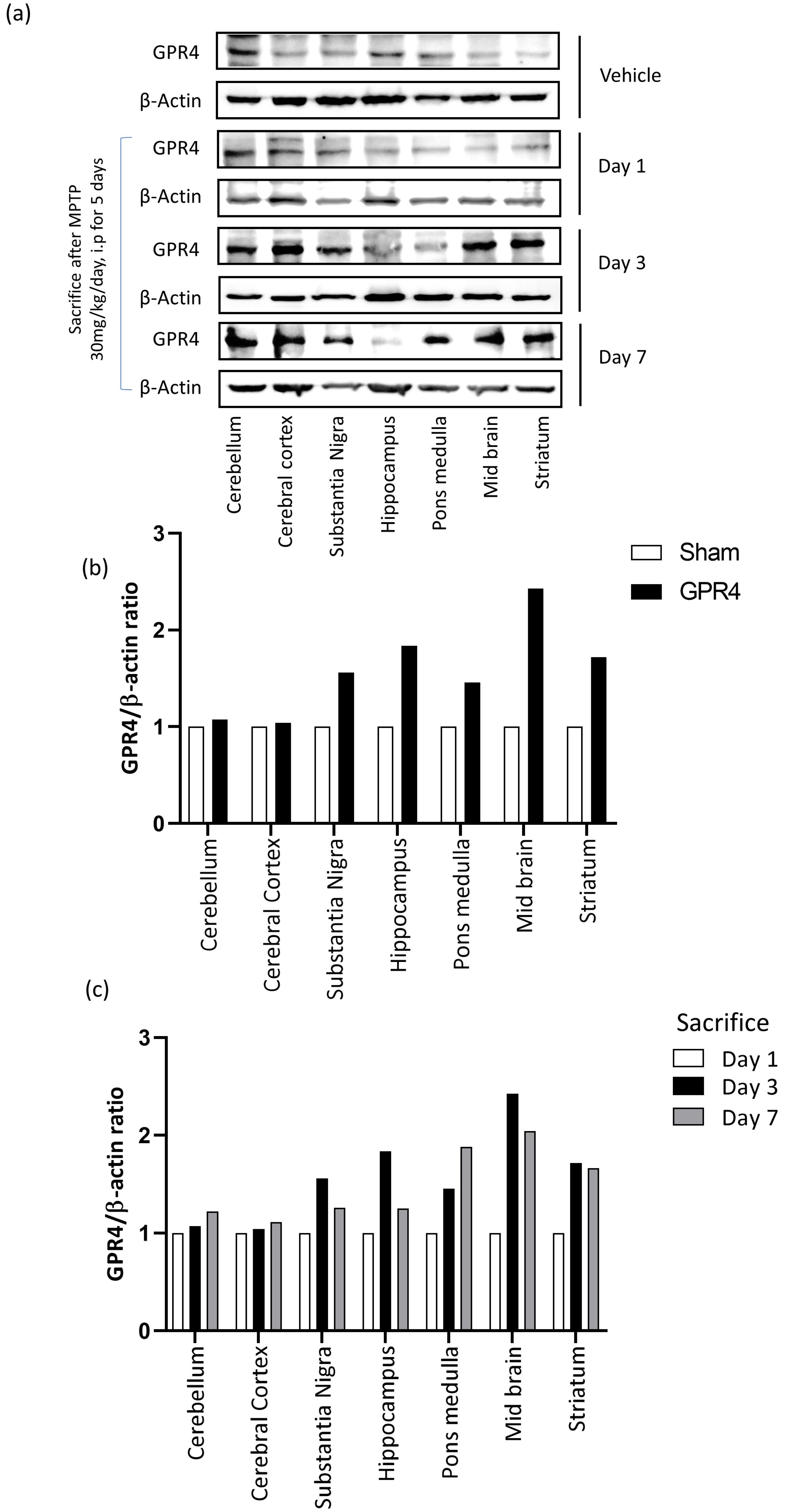

Supplementary figure S1:The effect of sub chronic administration MPTP, on the GPR4 Protein expression in different regions of mice brain. MPTP was treated (30mg/kg/day) for 5 days. Mice were sacrificed and tissue of different brain region was collected after 1, 3 and 7 days of last MPTP injection. (a). GPR4 protein expression in different regions of brain at different days after MPTP administration. (b). Densitometric analysis of the immunoblot data. β Actin ware used as an internal control.
